# Zebrafish as a Model Organism for the Development of Drugs for Skin Cancer

**DOI:** 10.3390/ijms18071550

**Published:** 2017-07-18

**Authors:** Fatemeh Bootorabi, Hamed Manouchehri, Reza Changizi, Harlan Barker, Elisabetta Palazzo, Annalisa Saltari, Mataleena Parikka, Carlo Pincelli, Ashok Aspatwar

**Affiliations:** 1 Biosensor Research Center, Endocrinology and Metabolism Molecular-Cellular Sciences Institute, Tehran University of Medical Sciences, 14114 Tehran, Iran; fatemeh.bootorabi@gmail.com; 2 Department of Aquaculture, Babol Branch, Islamic Azad University, 47134 Babol, Iran; hd_manuchehri@yahoo.com (H.M.); rech76ir@gmail.com (R.C.); 3 Faculty of Medicine and Life Sciences, University of Tampere, 33014 Tampere, Finland; harlan.barker@uta.fi; 4 Laboratory of Cutaneous Biology, Department of Surgical, Medical, Dental and Morphological Sciences, University of Modena and Reggio Emilia, 41100 Modena, Italy; elisabetta.palazzo1@gmail.com (E.P.); annalisax85@alice.it (A.S.); carlo.pincelli@unimore.it (C.P.); 5 Faculty of Medicine and Life Sciences, University of Tampere, Oral and Maxillofacial Unit, Tampere University Hospital, 33014 Tampere, Finland; mataleena.parikka@uta.fi

**Keywords:** melanoma, squamous cell carcinoma, inhibitor screening, transgenic zebrafish, skin cancer, drug development

## Abstract

Skin cancer, which includes melanoma and squamous cell carcinoma, represents the most common type of cutaneous malignancy worldwide, and its incidence is expected to rise in the near future. This condition derives from acquired genetic dysregulation of signaling pathways involved in the proliferation and apoptosis of skin cells. The development of animal models has allowed a better understanding of these pathomechanisms, with the possibility of carrying out toxicological screening and drug development. In particular, the zebrafish (*Danio rerio*) has been established as one of the most important model organisms for cancer research. This model is particularly suitable for live cell imaging and high-throughput drug screening in a large-scale fashion. Thanks to the recent advances in genome editing, such as the clustered regularly-interspaced short palindromic repeats (CRISPR)/CRISPR-associated protein 9 (Cas9) methodologies, the mechanisms associated with cancer development and progression, as well as drug resistance can be investigated and comprehended. With these unique tools, the zebrafish represents a powerful platform for skin cancer research in the development of target therapies. Here, we will review the advantages of using the zebrafish model for drug discovery and toxicological and phenotypical screening. We will focus in detail on the most recent progress in the field of zebrafish model generation for the study of melanoma and squamous cell carcinoma (SCC), including cancer cell injection and transgenic animal development. Moreover, we will report the latest compounds and small molecules under investigation in melanoma zebrafish models.

## 1. Introduction 

Zebrafish is a small vertebrate tropical fish that has recently emerged as one of the most useful models for studying human diseases, including cancers. In fact, zebrafish displays high fecundity and is suitable for genetic manipulation and both reverse and forward genetic studies. Thanks to its relatively low cost of use, the zebrafish is ideal for large-scale screening approaches and allows both chemical and genetic screening to identify genes and pathways underlying diseases, as well as phenotypic screening for the discovery of new drugs [1,2]. The compounds, drugs or small molecules, can be added directly to the water environment of the zebrafish [3]. Moreover, given the high genetic and physiological similarities with humans, zebrafish can be used as a useful and cost-effective vehicle for high-throughput screening (HTS) [2,4,5,6].

The growing interest in this model is derived from the optical transparency of zebrafish embryos and larvae, as well as their fast development ex utero. In fact, zebrafish are capable of fertilizing 200–300 eggs every 5–7 days and have an equivalent longevity and generation time to mice (3–5 months). Zebrafish embryos rapidly develop ex utero, and less than one week is required for the development of the digestive, nervous and cardiovascular organ systems [7,8,9,10,11,12]. This property allows for faster study of the physiological and pathological mechanisms by a direct live-cell imaging in vivo. For these reasons, zebrafish has become an important model for human diseases. Moreover, the high level of similarity between human and zebrafish larvae in terms of the genetics and physiology of the innate immune system promotes special investigation on cancer research [1]. Zebrafish is a suitable model for tumor induction by the use of several methods, such chemical treatments [13], genetic knockout [14], gene overexpression [15] and xenotransplantation [16]. Zebrafish has been used for the study of different type of cancers, such as skin cancer [14,15] pancreatic cancer [16], breast cancer [17], leukemia [18], glioma [19] and lung cancer [20]. 

Skin cancer, which includes melanoma and squamous cell carcinoma (SCC), represents the most common type of cutaneous malignancy worldwide, and its incidence is expected to rise in the near future [21]. Melanoma is the deadliest form of skin cancer, with respect to all skin cancer-related deaths, and has a mortality rate of 80% [22,23]. Melanoma arises from melanocytes, the pigmented epidermal cells responsible for the production of melanin. In the early stages, melanoma is confined to the epidermis (radial growth phase (RGP) melanoma) and can be removed by surgical excision [24]. These types of melanomas are usually associated with a good prognosis. On the contrary, in the next stages of tumor progression, melanoma cells invade the subcutaneous tissues (vertical growth phase (VGP) melanoma) and eventually progress toward the metastatic phase. At this stage, few therapeutic options are available, and melanoma frequently relapses and becomes incurable [6,24]. 

Cutaneous SCC (cSCC) mostly derives from alterations within the epidermal homeostasis caused by ultraviolet exposure and relies on uncontrolled proliferation of the epidermal cells, namely keratinocytes [25]. cSCC is the second most frequent type of non-melanoma skin cancer and represents 20% of all skin malignancies [25,26]. Although in situ SCCs are curable by surgical excision, metastatic SCCs are responsible for the majority of deaths due to non-melanoma skin cancers [21]. As a keratinocyte-derived epithelial tumor, head and neck squamous cell carcinoma (HNSCC) arises in several places in the head and neck region, such as oropharynx and laryngopharynx, and is very common worldwide, representing 4% of all cancers in the United States [23]. In particular, oral squamous cell carcinoma (OSCC) accounts for 24% of HNSCC with a mortality rate of 200,000 cases each year [26,27]. Despite different therapeutic options, such as radio or chemotherapy or surgery, the five-year survival rate is approximately 50% [26,28,29].

Given the significant impact of melanoma and SCC, on a world-wide scale, the development of new tools to study these pathologies and their treatment represents an important task. In the present review, we describe the zebrafish as a powerful in vivo model for skin cancer (melanoma and SCC) and summarize the advantages of drug screening in skin cancer research. We report in detail on the use of transgenic zebrafish to study melanoma development, progression and treatment [30,31] and the use of zebrafish embryos to evaluate potential targets and compounds for SCC [22,25]. 

### 1.1. Zebrafish as a Model Organism for Pharmacological and Toxicological Screening

**Figure 1:**
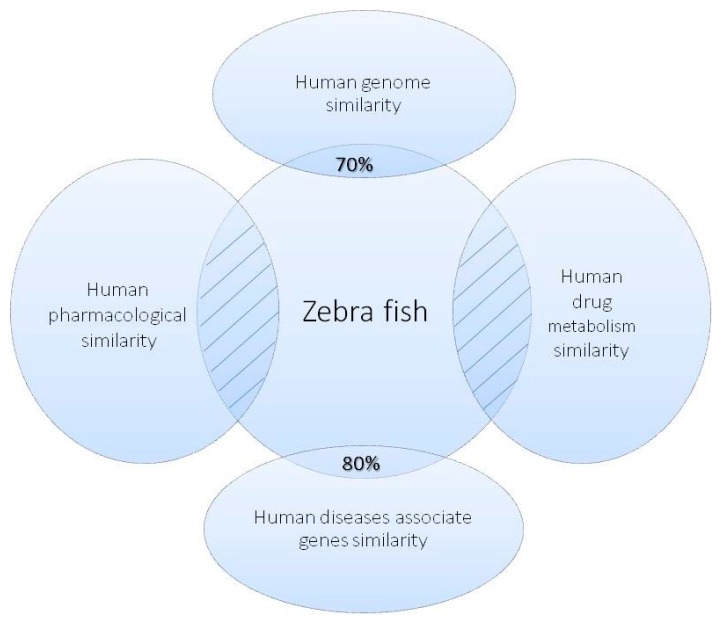
Zebrafish as a relevant model for human disease and cancer therapy. The zebrafish genome shows up to 80% similarity with human disease-associated genes. Moreover, thanks to a well-conserved physiology, the pharmacological behavior and metabolism of several drugs have been screened in zebrafish with effects similar to humans.

In recent years, research has begun to develop an understanding of how zebrafish can be used as an in vivo model for human diseases, in terms of targets for therapy, physiological similarity, drug metabolism, drug safety and toxicity and pharmacology [2] (Figure 1). The zebrafish genome shares over 70% similarity with the human genome, and over 80% of known human disease genes, including oncogenes and tumor suppressor genes, have orthologues in zebrafish [32]. Several pathways are also well conserved between human and zebrafish allowing targeting of a specific biological process [32]. Therefore, the results obtained by using zebrafish models for drug screening can be particularly relevant for human disease and cancer therapy.

Cell-based assays mostly provide limited results and information about the absorption, distribution, metabolism and toxicity of the screened molecules, compounds and drugs. By contrast, screening in zebrafish overcomes this problem; moreover, there are physiological similarities in the blood brain barrier [10], endothelial cells [33], endothelial cells and immunological responses [34], giving fast data related to the pharmacological characteristics [35,36,37]. When injected with a compound, either a drug or small molecule, zebrafish and mammals present overlapping physiologic responses, such as the induction of metabolites, enzyme activity against different antigens and upon oxidative stress conditions [4,9].

**Figure 2:**
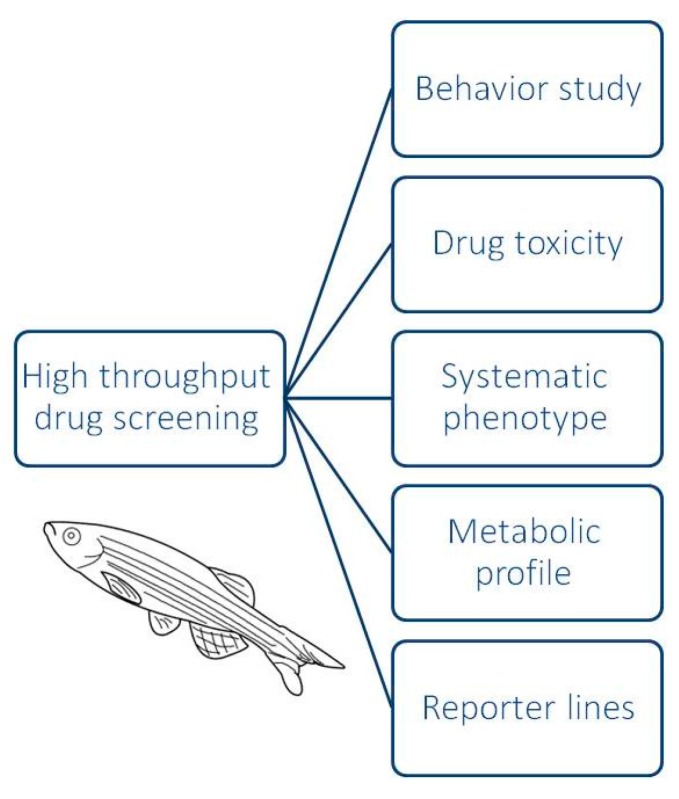
Zebrafish model for high-throughput drug screening. Zebrafish is a valuable tool for high-throughput screening assays. In terms of toxicity, zebrafish have been used to evaluate a specific organ or behavior with respect to toxicity. In drug screening, zebrafish is also established as precious platform to multiple systemic phenotype studies simultaneously with metabolic profiles and toxicity reporter lines.

A number of the biological properties of zebrafish provides advantages for drug screening. Early-stage zebrafish embryos have a transparent body, making it relatively easy to collect numerous data using a high-quality imaging after treatment. Moreover, given that a single female can lay 10,000 eggs per annum [38], it is possible to reach high throughput (1000–10,000) or more assays per day, as only space becomes a limiting factor [39] (Figure 2). For this reason, zebrafish embryos have been proposed as the in vivo animal model that could bridge the gap between cell-based assays and biological validation of a compound. Moreover, the presence of the chorion, an acellular envelope made of three intercrossing layers that surround the embryo during development, allows some materials to easily pass through to the embryo via passive diffusion [40]. This aspect is relevant in terms of physical-chemical properties of the compounds that could be tested by zebrafish models, by affecting the efficacy of the treatments or the level of dose-specific toxicity. 

Because of the transparency of zebrafish embryos and larvae and the possibility to conduct morphological observation of various organs and anatomic structures during development [8,10,11,12,41], zebrafish has assumed an important role in toxicology research, including ecotoxicology and developmental studies. The greatest advantage of zebrafish in toxicology studies is the low cost with respect to rodents and the possibility to perform analysis with a larger number of compounds at the same time. In this respect, zebrafish can be utilized for large-scale screening of compounds/libraries and drugs, as it may give faster results. In fact, hundreds of different hits from a primary HTS can be tested to eliminate the toxic compounds and then prioritizing those that will be utilized in further assays and drug development. This implicit counter-screen for toxicity is an integral part of disease suppressor screens in the organism [2]. 

In addition, the transplantation of human cancer cells into zebrafish to generate xenograft models represents a powerful tool to monitor cancer proliferation, tumor angiogenesis, metastasis and drug response in real time. 

The use of fluorescent probes or reporter genes during toxicological or cancer-related studies gives the opportunity to perform live-imaging for both transgenic and xenotransplantation models. For example, melanoma cells can be stained with Vybrant Cell-Labeling Solution or (5(6)-Carboxyfluorescein *N*-hydroxysuccinimidyl ester (CFSE) dye before being microinjected in the yolk [42]. To identify the role of Raf and phosphoinositide 3-kinase (P13K) signaling pathways in melanoma cells, a Green Fluorescent protein (GFP) reporter gene was used under the control of the promoter of the melanoma cell specific gene, microphthalmia-associated transcription factor (*mitf*) [24]. Similarly, in order to investigate the implication of Extracellular signal-Regulated kinases (ERK1/2)-Mitogen-Activated Protein kinases (MAPK) signaling pathways in the development of uveal melanoma, transgenic embryos were labeled with GFP and coupled to the GNAQQ209P vector in order to express this oncogene in melanocytes [30]. Moreover, inter-segmental blood vessel cells were labeled with GFP in transgenic embryos for the study of melanoma cell adhesion [15].

Nevertheless, as a consequence of the complexity and inherent differences of mammals, it is important to highlight that zebrafish can never replace rodents in the later phases of drug discovery, but may be complementary to rodent or cell-based assays at earlier stages [43]. Indeed, clinical trials using several murine models for human inflammatory pathologies fail to show a significant success rate in humans [44]. Hence, understanding the advantages, disadvantages and limitations of mammalian models helps to choose the best model for pharmacological studies according to the specific target. 

### 1.2. Phenotype-Guided Drug Discovery

Despite the achievement of significant progress in drug discovery, the identification of new therapeutic targets for the treatment of human diseases remains a big challenge in science [45]. 

While previous studies were restricted to invertebrate model organisms, such as *Caenorhabditis elegans*, or to in vitro cell-based assays, because of extensive labor and cost requirements, the use of zebrafish has opened a new avenue [45]. Thanks to the random mutagenesis strategy, a greater range of phenotypes resembling human diseases can be investigated in comparison to other vertebrates such as mice and rats. In contrast to target-based approaches, phenotype-guided drug discovery aims to carry out a large chemical screening by combining HTS with animal models for a human disease. Hence, using the phenotype-guided drug discovery, it is possible to identify an effective compound by looking at phenotype alteration in the whole organism, independently of the specific target. Therefore, this approach improves the development of new drugs and, at the same time, allows the identification of the pathways that are implicated in the development or progression of the specific disease [6]. Two examples of the use of zebrafish for phenotype-based drug discovery are given by the screening of chemical molecules as suppressors of two specific genetic mutations: (1) gridlock and (2) Crumbs homolog (*crb*) mutations [45]. Gridlock mutants, which present a vascular defect due to a mutation in the *hey2* gene, have been treated with a library of 5000 small molecules to revert the disease phenotype. From the screening, two compounds, named GS4012 and GS3999, have been identified. These molecules activate the vascular endothelial growth factor (VEGF) pathway to rescue the pathological phenotype. The second mutant harbors a mutation in the Myb proto-oncogene protein also known as transcriptional activator Myb (*myb*) gene, which leads to genome instability and defects in the cell-cycle. A library of 16,320 compounds was screened in crb embryos, allowing the discovery of three different classes of suppressors of the crb mutant phenotype. The possibility to report human disease alleles in zebrafish further supports the high impact of this method in drug discovery [46,47,48,49]. 

## 2. Zebrafish as a Model Organism to Study SCC

The zebrafish model has been recently used to identify key molecules in cSCC and HNSCC [23], as well as compounds for SCC target therapy [50].

Cichon and co-workers found that the tyrosine kinase receptor Axl, which is highly expressed in SCC where it promotes cell survival, is implicated in tumor formation in the cSCC xenograft model in zebrafish [51]. Indeed, Axl deletion in cSCC cells reduced tumor mass, after cell injection in the yolk sac of one-day-old embryos, thus indicating that Axl is a potential therapeutic target for SCC. Using a similar approach, the role of type VII collagen (Col7) in cSCC tumor growth and angiogenesis was also evaluated. Mutation in the *COL7A1* gene is responsible for dystrophic epidermolysis bullosa (RDEB), an inherited blistering disorder that predisposes to the development of an unexplained aggressive SCC [25]. Recombinant type VII collagen (hrCol7) was able to reverse SCC angiogenesis in the xenograft model [25]. 

The tyrosine kinase discoidin domain receptor 2 (DDR2), which has a role in cell proliferation, adhesion, differentiation and invasion, is implicated in HNSCC [21]. DDR2 has been reported to be inhibited by dasatinib, a Food and Drug Administration (FDA)-approved inhibitor of Abelson murine leukemia viral oncogene homolog, Proto-oncogene tyrosine-protein kinase (ABL, SRC) and c-Kit [21]. Von Massenhausen and collaborators analyzed the functional role of DDR2 in an in vivo xenograft model with or without dasatinib treatment and demonstrated that DDR2 inhibition blocked HNSCC cell migration and invasion [21]. Their research indicates that dasatinib can be potentially used as a tyrosine kinase inhibitor in DDR2-positive HNSCC patients. 

Zebrafish embryos have also been used to evaluate the effect of Flotillin-1 overexpression in KB cells (a subline of the KERATIN-forming tumor cell line HeLa), which are an OSCC cell line [26]. Flotillin-1 is a component of the lipid rafts and plays an essential role in cell adhesion, cell morphology and protein secretion [26]. Zebrafish embryos were injected with fluorescent-labeled tumor cells overexpressing Flotillin-1 to analyze tumor metastasis. The results demonstrated that the expression of Flotillin-1 increases the cell growth and motility of KB cells [26]. 

The motility of OSCC was also targeted by the use of the neutralizing monoclonal antibody NZ-1 and lectin (MASL) against podoplanin (PDPN)-expressing OSCC cells in zebrafish. This molecule is a transmembrane receptor that promotes tumor cell motility in OSCC, and it might be used as a chemotherapeutic target for primary and metastatic cancers [6]. 

Among the compounds being evaluated in zebrafish for SCC, triazine compound S06 reduces OSCC invasion. Its mechanism of action is targeting of the chaperon heat-shock protein 90 (Hsp90); thus, inhibiting carcinoma-associated fibroblast (CAF)-derived proinvasive chemokinases by S06 is capable of inhibiting tumor cell migration in a zebrafish xenograft model at 48 h post-fertilization [50]. The marine microbial extract luminacin was evaluated in zebrafish embryos in terms of anti-tumor activity in HNSCC [23]. Luminacin treatment of cancer cells was able to inhibit growth and cancer progression by promoting autophagy of HNSCC cell lines.

Taken together, these works well support the use of zebrafish as a model organism for the analysis of the key players in SCC development and progression, as well as for drug screening and toxicity assays. 

## 3. Zebrafish as Model Organism for Melanoma Research 

While the importance of the activation of oncogenes and inactivation of tumor suppressor genes in tumor formation is well appreciated, our understanding of the early events of cancer initiation remains limited. The mechanisms that enable a subpopulation of tumor cells to complete the conversion to a malignant state among a larger group of cancer-prone cells (described as a “cancerized field”) is still unclear [52]. To better understand the mechanisms underlying tumor initiation, in melanoma research, zebrafish can be used as an excellent tool, through the use of xenograft [42,53] and transgenic models [30,54].

The main advantages of xenotransplantation are live cell imaging and the lack of the adult immune system, which is completely functional only at 28 days of development. The injection of fluorescent-labelled melanoma cells into zebrafish larvae enables the study of cancer angiogenesis and tumor cell spread. The early phases of melanoma progression and the phenotype switching toward metastatic behavior have been recently investigated in zebrafish through the study of the neurotrophin receptor CD271 (p75NTR) [42]. CD271 is variably expressed in melanoma cells, showing higher levels in primary tumors compared to the metastatic tumors derived from the same patient. The injection of melanoma cells after CD271 silencing or overexpression in transparent larvae revealed that CD271 absence is associated with a higher number of metastases in zebrafish [42]. 

Since adult zebrafish are no longer transparent, in order to visualize melanoma cells by fluorescent gene expression, a transparent adult fish, named *casper*, has been created by White et al. [55]. 

Alternatively, the study of melanoma development can be performed in transgenic zebrafish derived from the expression of oncogenes or from mutations in tumor suppressor genes induced with *N*-ethyl-*N*-nitrosourea (ENU). ENU is a powerful mutagen with alkylating activity used as a highly efficient strategy to induce random mutations in the genome of zebrafish. This approach consists of exposing zebrafish to repeated treatments with ENU to generate point mutations in pre-meiotic germ cells [53]. This treatment causes all possible base pair changes, such as nonsense, splicing and missense mutations. ENU has been successfully used to create a series of knockout zebrafish [56]. Given that the phenotype associated with alterations in the zebrafish development was easily noticeable, ENU was used to investigate the role of mutations in embryonic patterning and development. However, some disadvantages limited the use of this random mutagenesis approach, such as the generation of heterozygous mutants that hamper the identification of recessive inherited phenotypes. By using this approach, three mutants named “*young*” (*Yng*), “*perplexed*” (*plx*) and “*confused*” (*cfs*) affected by defects in retinal lamination have been identified [57]. More recently, the clustered regularly-interspaced short palindromic repeats (CRISPR)/CRISPR-associated protein 9 (Cas9) system has emerged as a new genome editing tool to induce mutations in specific genes within organisms [58]. CRISPR comprises segments of DNA containing short, repetitive base sequences. These sequences play a role in a bacterial immune system. The CRISPR/Cas9 strategy is based on delivering the Cas9 DNA nuclease with a guide RNA (gRNA) into a cell to cut the genome at a specific location. A series of successful studies used this technology to evaluate the role of specific genetic perturbations on the phenotype of zebrafish. For example, it has been used to study the effect of MMP21 knock-out, revealing a role in cardiac defects and alteration of Notch signaling [59]. Moreover, this genome editing system has a great impact in cancer research, as it permits tissue-specific modelling of human cancer, such as melanoma [60,61,62]. 

Melanoma frequently originates due to mutation in the *BRAF* and *NRAS* genes (43% and 30% of cases, respectively) [63]. Because BRAF acts as a serine/threonine kinase and becomes activated by a somatic point mutation, it plays an important role as a target for drug development in malignant melanoma [64]. The first melanoma zebrafish model carrying the V600E mutation was obtained by placing the human *BRAF^V600E^* gene under the control of the *mitf* (microphthalmia-associated transcription factor) promoter, a transcription factor involved in the lineage-specific pathway of melanocytes [65]. The *BRAF^V600E^* mutation is associated with melanoma development; however, the concomitant loss of function of the p53 gene is needed to make melanocytic naevi progress to cancer [64]. Even after the generation of an extensive “cancerized field”, with all melanocytes harboring both *BRAF^V600E^* and *p53-/-* mutations in zebrafish, only one to three melanomas will develop and only after several months [65]. This lesser number and time frame indicate that other molecular alterations and pathways play an important role in tumor formation. By screening the genes implicated in the rapid onset of melanoma, Zon and colleagues found that the overexpression of histone-lysine *N*-methyltransferase (SETDB1) can accelerate the onset and invasion of melanoma [4]. High levels of SETDB1 are common in human melanoma, indicating that chromatin remodeling may be critical in melanoma progression due to changes in gene regulation, such as subset of homeotic genes (*hox*) genes [66]. 

Transgenic zebrafish carrying RAS mutations for the study of melanoma have also been created [24]. Expression of the HRAS^G12V^ protein, under the *mitfa* promoter, in melanocyte progenitors induces a hyper-pigmented phenotype and an abnormal growth of melanocytes in the larvae; ultimately driving melanoma formation in the adult fish [67]. Moreover, elevated activity of RAC, a subfamily of Rho GTPases, often associated with melanoma in humans, can accelerate the progression of HRAS^V12^-driven malignant melanoma [68]. Zebrafish *BRAF^V600E^* models have some limitations, such as the time needed for spontaneous tumor formation and the different number of mutations as compared to human melanomas [69]. On the other hand, *HRAS^V12^* mutations accelerate melanoma formation and enable melanoma to be visualized at the earliest stages in zebrafish [7,24,70]. 

Finally, the zebrafish model allows the study of genes involved in the lineage-specific pathway for melanocyte development and how their misregulation is responsible for melanoma initiation [71]. For this purpose, a temperature-sensitive zebrafish model harboring a mutation in the *mitf* gene (*mitfa*^vc7^) has recently been created [71,72,73]. 

## 4. Compounds and Inhibitors for Melanoma Treatment

Previous studies have shown that *mitf* is a potential therapeutic target for melanoma treatment [67,74,75]. MITF plays a critical role in the development of the melanocytic lineage [76,77] and regulates genes associated with melanogenesis, cell differentiation, proliferation and survival [78,79,80] by targeting BCL-2 (B-cell leukemia/lymphoma 2) [81], ML-IAP (melanoma inhibitor of apoptosis) [82,83], HIF1a (hypoxia-inducible factor 1a) [84] and MET [85]. Moreover, MITF can regulate the expression of CDKN1A [86,87], which encodes for p21, an inhibitor of the cell-cycle. Through screening of a small molecule library containing 2000 compounds, SKLB226 has been identified as a specific suppressor of *mitf* expression, subsequently able to inhibit the viability and migration of melanoma cells [88]. In addition, the evaluation of SKLB226 action in zebrafish showed its ability to block pigmentation and cell migration in vivo [89]. Levi et al. used zebrafish embryos to test a library of 6000 compounds identifying two molecules (12G9 and 36E9) able to inhibit melanocyte development by targeting *mitf* [90]. 12G9 and 36E9 inhibited pigmentation of both skin and retinal pigment epithelium [91] and induced apoptosis of melanocytes [76]. In particular, the compounds impaired the proliferation and differentiation of melanoblasts and induced a reduced number of *mitfa* and *dct* (dopachrome tautomerase, a protein required for the synthesis of eumelanin) positive cells [91]. In addition, among the sulfur-containing molecules, 4 (phenylsulfanyl) butan-2 reduces the expression of melanin synthesis-related proteins and exhibits a good safety profile. This compound induces a remarkable suppression of melanogenesis in zebrafish after 48 h. In addition, its ability to repress in vivo pigmentation could have significant applications in the cosmetic field [91]. Furthermore, this whitening substance is safe and effective, and it could be used to prevent hyper-pigmentation [91].

Zebrafish embryos carrying *BRAFV^600E^* expression have been used for the study of the neural crest-derived cell lineage [92]. In particular, transgenic mitf-BRAF^V600E^; p53/zebrafish embryos have been created for the evaluation of early transcriptional activity within melanoma pathogenesis and to provide a model for chemical genetic screening in the context of melanoma therapy. 

Inhibitors of dihydroorotate dehydrogenase (DHODH), such as leflunomide (lef), are able to reduce the ability of self-renewal of neural crest cells and target the activity of those genes required for neural crest development and melanoma tumor growth [93], including *mitf* and *myc* targets [92]. Research data suggest that lef would be more effective in combination with a BRAFV^600E^ inhibitor and may help to overcome resistance to BRAFV^600E^ inhibitors. Current goals involve the identification of more lineage-specific suppressors in zebrafish embryos that can be generalized to other cell types, with less side effects and toxicity and direct relevance to human cancer.

**Figure 3:**
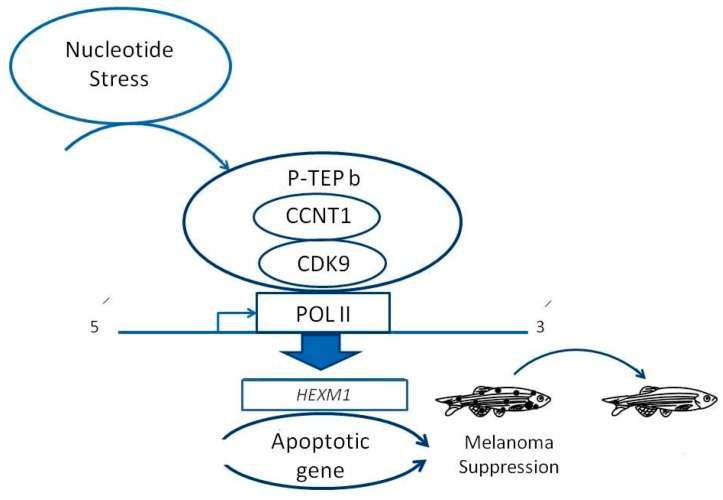
The tumor suppressor hexamethylene bisacetamide inducible 1 (*HEXIM1)* gene inhibits melanoma in the zebrafish model. *HEXIM1* plays an important role as a melanoma tumor suppressor in response to nucleotide stress. *HEXIM1* forms a complex with positive transcription elongation factor (P-TEFb) in order to inhibit the kinase to initiate transcription elongation at tumorigenic genes. Alteration of gene expression, in parallel with anti-tumorigenic RNAs binding to *HEXIM1*, favors the “anti-cancer” gene expression. Pol II: DNA polymerase II; CDK9: cyclin-dependent kinase; CCNT1: Cyclin-T.

The inhibition of DHODH by lef ablates zebrafish neural crest development and suppresses melanoma growth by blocking the transcriptional elongation of key genes, such as *crestin* and *mitfa* [92]. HEXIM1 (hexamethylene bisacetamide inducible 1) works as a general RNA polymerase II transcription inhibitor, and its expression regulates gene expression during differentiation. HEXIM1 suppresses melanoma tumors in response to nucleotide stress, and its overexpression in a zebrafish model suppresses melanoma formation [94]. *HEXIM1* knockdown by morpholinos partially rescued *crestin* and *mitfa* expression in lef treated zebrafish embryos [94]. This mechanism involves control of the elongation phase of RNA transcription and regulates gene expression during differentiation [93]. The function of *HEXIM1* in melanoma was investigated after its overexpression and deletion in a zebrafish model, revealing its role as a tumor suppressor gene. Other cell types may be susceptible to different cellular stresses and respond in a similar manner by activating *HEXIM1* to help the cell repair or induce cell death. Finding such dependencies in other cancers could lead to the development of new cancer therapies using the zebrafish model (Figure 3) [62,95]. 

**Figure 4:**
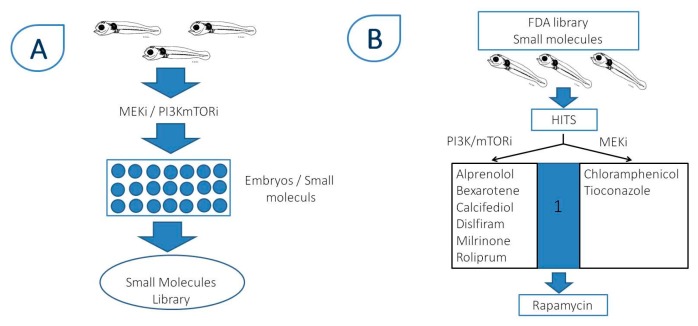
Drug development and inhibitor screening using selected MEKi and PI3K/mTOR inhibitors. Zebrafish plays an important role for the screening of compounds targeting MEK/ERK and PI3K/mTOR pathways, both alone and in combination. (**A**) Example of screening of the FDA library molecules using zebrafish embryos; (**B**) steps showing hit selection after the screening procedure with different drugs and drug dose response using the melanin assay. At the end of this process, 11 hits were detected to be further evaluated in cell culture. MEKi: mitogen-activated protein kinases inhibitor; PI3K: phosphoinositide 3-kinase; mTOR: mechanistic target of rapamycin.

A transgenic zebrafish melanoma model was developed by coupling the zebrafish *mitfa* promoter with the human oncogenic HRASG12V [96]. These transgenic embryos developed melanocyte hyperplasia with the induction of RAS-RAF-MEK-ERK and RAS-PI3K-AKT-mTOR signaling pathways. In this context, the zebrafish model was useful for the screening of compounds directed against mitogen-activated protein kinases, extracellular signal-regulated kinases (MEK/ERK) and PI3K/mTORi pathways, alone or in combination. Rapamycin, a well-known mTOR inhibitor, is considered as one of the strongest drugs in the field [97]. It was suggested that rapamycin analogs might also improve access to the kinase active site of ATP competitive inhibitors. The ability of rapamycin analogs to co-operate with MEKi to suppress melanoma cell growth was previously observed. Because of the efficacy of cocktails of inhibitors observed with HTS assays in vitro, clinical trials using rapamycin analogs combined with MEKi or PIK3K/mTORi are currently underway [98] (Figure 4).

## 5. Conclusions and Future Directions

As zebrafish research develops further, new knowledge and tools are becoming available that will significantly impact the range and quality of in vivo chemical screening. Recent and future advances in zebrafish research will certainly have an impact that will include improvements in data acquisition (particularly imaging), increased automation capability, advances in genetic manipulation and the development of new phenotypic endpoints. A new era of combined therapy for metastatic melanoma is now on the way. To further address the development of resistance, clinical trials involving triple combination therapies are ongoing. In this respect, the combination of BRAF and MEK inhibitors with anti-ErbB3 antibodies, at least based on preclinical data, predicts a future clinical use.

Replacing the target-based drug discovery with the newly-introduced phenotype-guided discovery has opened an important gate for chemical and genetics screening. HTS in combination with zebrafish as an in vivo disease model represents a valuable tool for toxicity studies and small molecules screening. At the same time, a large number of molecules and compounds can be screened with the use of zebrafish in order to identify effective drugs and observe disease phenotypes.

The incidence of skin cancer is showing a rapid increase worldwide for several reasons, from unprotected exposure to the Sun to the aging of the population [68]. SCC and melanoma are malignant conditions of the skin for which the need for drug development is crucial, because of the number of patients with metastases and the poor outcome for advanced stages. In this context, zebrafish models give the opportunity to both explore new pathways and test new compounds or small molecules that could be used in clinical trials. Simultaneously, the effect on embryonic development and toxicity is rapidly available and allows optimized targets and doses. Therefore, the development of zebrafish models will help to bridge the gap between in vitro cell culture and in vivo mammalian models for a rapid pre-clinical drug development.
